# Comparing computational times for simulations when using PBPK model template and stand-alone implementations of PBPK models

**DOI:** 10.3389/ftox.2025.1518769

**Published:** 2025-02-19

**Authors:** Amanda S. Bernstein, Paul M. Schlosser, Dustin F. Kapraun

**Affiliations:** ^1^ Oak Ridge Institute for Science and Education, Oak Ridge, TN, United States; ^2^ Center for Public Health and Environmental Assessment, Office of Research and Development, US Environmental Protection Agency, Research Triangle Park, NC, United States

**Keywords:** PBPK model, template model, risk assessment, pharmacokinetics, computational timing

## Abstract

**Introduction:**

We previously developed a PBPK model template that consists of a single model “superstructure” with equations and logic found in many physiologically based pharmacokinetic (PBPK) models. Using the template, one can implement PBPK models with different combinations of structures and features.

**Methods:**

To identify factors that influence computational time required for PBPK model simulations, we conducted timing experiments using various implementations of PBPK models for dichloromethane and chloroform, including template and stand-alone implementations, and simulating four different exposure scenarios. For each experiment, we measured the required computational time and evaluated the impacts of including various model features (e.g., number of output variables calculated) and incorporating various design choices (e.g., different methods for estimating blood concentrations).

**Results:**

We observed that model implementations that treat body weight and dependent quantities as constant (fixed) parameters can result in a 30% time savings compared with options that treat body weight and dependent quantities as time-varying. We also observed that decreasing the number of state variables by 36% in our PBPK model template led to a decrease of 20–35% in computational time. Other factors, such as the number of output variables, the method for implementing conditional statements, and the method for estimating blood concentrations, did not have large impacts on simulation time. In general, simulations with PBPK model template implementations of models required more time than simulations with stand-alone implementations, but the flexibility and (human) time savings in preparing and reviewing a model implemented using the PBPK model template may justify the increases in computational time requirements.

**Conclusion:**

Our findings concerning how PBPK model design and implementation decisions impact computational speed can benefit anyone seeking to develop, improve, or apply a PBPK model, with or without the PBPK model template.

## Introduction

Physiologically based pharmacokinetic (PBPK) models describe the absorption, distribution, metabolism, and elimination of a chemical in an organism following exposure. These models can provide estimates of time course concentrations of a chemical in the blood and specific body tissues. When implementing a PBPK model using a computer, one must make several decisions that affect the ease of implementing and using the model and the performance of model simulations, including computational speed. One must first decide which platform or language to use for model implementation. Like other models that consist of systems of ordinary differential equations (ODEs), PBPK models can be implemented using interpreted languages (like R, Python, or Matlab) or compiled languages (like C or Fortran). Many PBPK modelers work primarily with interpreted languages (even though this results in slower simulations) because using such languages makes it easier to encode and iteratively develop models. Another implementation option involves combining the convenience of interpreted languages and the speed of compiled languages. In one such approach, modelers use R and MCSim together: first, they use the MCSim model specification language (Bois, 2009), which is relatively easy for humans to read, to define parameters and encode differential equations in a single file. Using the MCSim “mod” tool, this model file can be translated to C and then compiled for use with simulation scripts written in R. Modelers usually choose an implementation language and platform based on their prior expertise, computational sophistication, financial resources, and other factors. However, the time needed to implement and run a model can certainly also be a factor in such decisions.

PBPK models are commonly used to perform dosimetric calculations in support of chemical risk assessments, but the models should undergo a rigorous quality assurance (QA) review prior to use (McLanahan et al., 2012). This includes evaluating the structure, purpose, and mathematical description of the model, as well as ensuring that the computer implementation is free from errors and can sufficiently reproduce experimental data (Clark et al., 2004; IPCS, 2010; McLanahan et al., 2012; U.S. EPA, 2020). Once a PBPK model has passed QA review, it may be considered suitable for application. In some cases, the assessor might use the model to perform just a few simulations, for example, to compute a dose metric or human equivalent dose for one or a few exposure scenarios with a single set of model parameters per species of interest. In other cases, however, many thousands of simulations may be required. Monte Carlo analyses, in which many simulations are performed for different sets of model parameters in order to quantify uncertainty and variability in model predictions, are becoming more common in PBPK modeling applications (Chiu et al., 2014; Krauss and Schuppert, 2016; Clewell et al., 2019). The computational time required for a single simulation can greatly impact the overall time required for a Monte Carlo analysis, which might involve hundreds of thousands of simulations (Hamra et al., 2013; Tsiros et al., 2019; Wedagedera et al., 2022).

**FIGURE 1:**
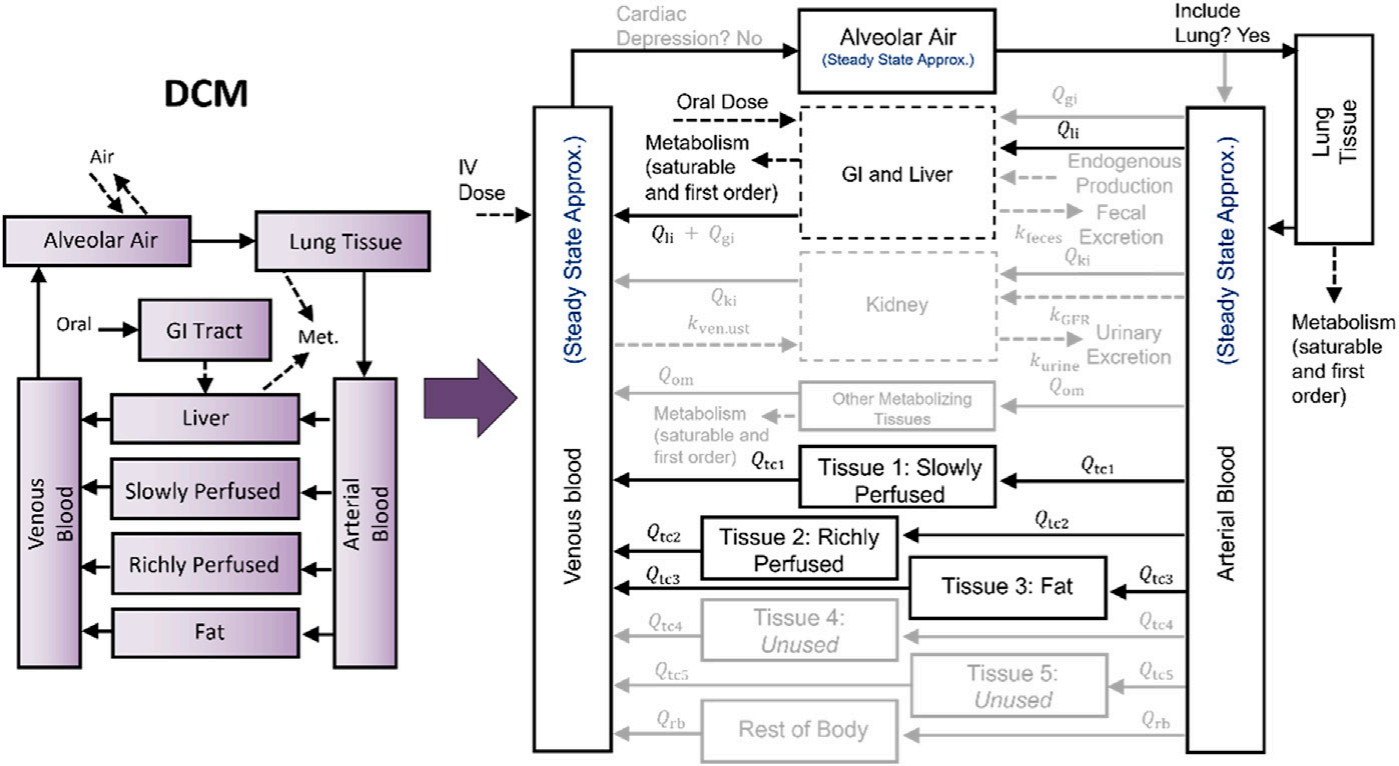
Mapping a PBPK model for dichloromethane (DCM) to the PBPK model template superstructure. The model template superstructure includes multiple tissue compartments and features that commonly appear in published PBPK models, so to implement a particular chemical-specific PBPK model using the PBPK model template, the desired structure must be mapped onto the model template superstructure by setting the appropriate parameters. Unused parameters are set to zero, and for unused compartments, the respective blood flows are set to zero. The mapping of the U.S. EPA (2011) PBPK model for DCM is shown here by using lighter colored arrows, boxes, and text for parameters and compartments that are “switched off” or set to zero.

Previously, we developed and described a PBPK model template that allows one to implement many different chemical-specific PBPK models using a single model “superstructure” with equations and logic commonly found in PBPK models (Bernstein et al., 2021; Bernstein et al., 2023). The template includes a wide variety of features allowing for the implementation of many different PBPK models, but in implementing any given chemical-specific PBPK model, only a subset of the available features are typically used. For example, Figure 1 illustrates how a PBPK model for dichloromethane (DCM) was mapped onto the PBPK model template superstructure, with darker and lighter rectangles and arrows indicating the features that are included and excluded, respectively. Source code and documentation for the most recently published version of the PBPK model template is available online (https://doi.org/10.23719/1527967). We implemented the PBPK model template using R and MCSim to balance ease-of-use with computational speed. The ability to write simulation scripts in R means that the template is relatively easy for PBPK modelers and risk assessors to use, while our specification of the mathematical details of the PBPK model template in the MCSim model-building language, which can be easily translated to C, takes advantage of the faster speed that can be achieved using compiled code. When PBPK models are implemented using the template, less time may be need for QA review because the PBPK model template equations, structure, and implementation source code have already undergone a thorough QA process. That is, when performing a QA review for a chemical-specific PBPK model that has been implemented using the template, one only needs to consider whether the equations and logic selected for inclusion are appropriate and to verify that the input spreadsheets used to set parameter values for the model and exposure information for the desired simulations have been correctly specified.

To give the model template the flexibility needed to implement a wide range of chemical-specific PBPK models, more compartments and options for modeling different processes are included than would be included in any stand-alone PBPK model implementation. Therefore, expressions for many unused quantities will typically be evaluated when performing a simulation for a given model using the PBPK model template. In light of this, one might expect that simulations performed using a template-implemented model would require significantly more time than those performed using a stand-alone model implementation. We explored the impact that different model implementation decisions have on computational speed, including the extent to which the computational time needed for template-implemented model simulations differs from that needed when using stand-alone model implementations.

There is limited literature currently available on the impact of PBPK modeling implementation decisions on computational speed of simulations. Several studies (Snowden et al., 2018; Bloomingdale et al., 2021; LeFew and El-Masri, 2012) considered how combining model compartments (which represent tissues or organs) to create a new model with fewer compartments (i.e., “lumping”) reduced computational time requirements. Bloomingdale et al. (2021) considered the development of a PBPK model focused on the brain for antibody therapeutics and showed that by combining tissue compartments together, they could reduce the number of differential equations in their model from 100 to 16 and achieve an improvement in simulation speed of approximately 11-fold. Similarly, Snowden et al. (2018) considered using lumping to reduce linked PBPK models describing the effects of drug administration and found they could decrease simulation time by up to 80% by doing so. Other studies have explored how high-throughput PBPK models can be used to quickly assess many chemicals and the reductions in simulation time associated with such models (Naga et al., 2022). However, none of these studies involved a systematic evaluation of how selection of model features impacts computational speed for model simulations.

## Methods

**FIGURE 2:**
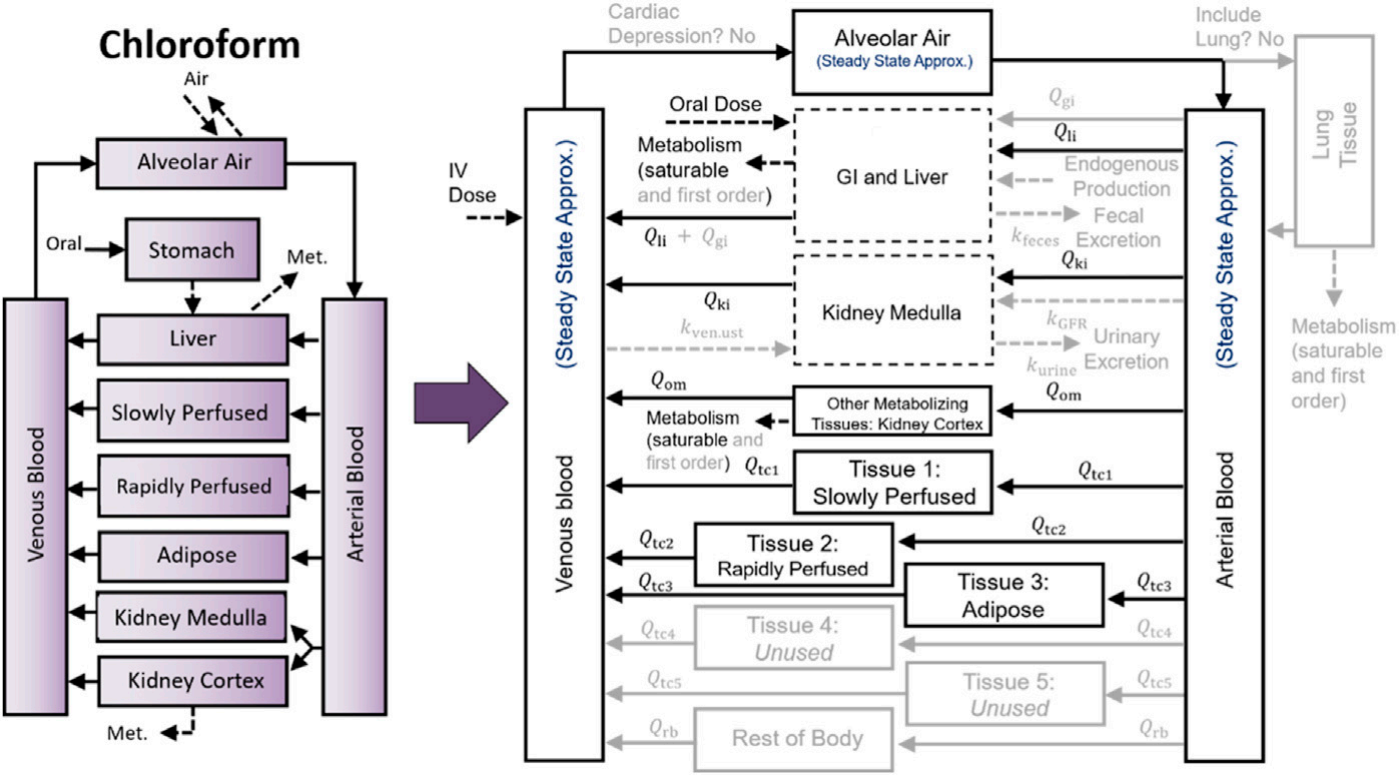
Mapping a PBPK model for chloroform (CF) to the PBPK model template superstructure. To implement the Sasso et al. (2013) PBPK model for CF, we mapped the existing model structure to the PBPK model template superstructure by setting the appropriate parameters. Unused parameters are set to zero, and for unused compartments, the respective blood flows are set to zero. The mapping of the published CF model to the model template superstructure is shown here by using lighter colored arrows, boxes, and text for parameters and compartments that are “switched off” or set to zero.

We performed various timing experiments using two chemical-specific PBPK models–one for DCM (U.S. EPA, 2011) and one for chloroform (CF) (Sasso et al., 2013) – and four different exposure scenarios–constant continuous oral, periodic oral, constant continuous inhalation, and periodic inhalation exposures (U.S. EPA, 2011; Sasso et al., 2013). We used PBPK model template implementations of the DCM and CF PBPK models (depicted in Figures 1, 2, respectively) that have been described in detail by Bernstein et al. (2023), as well as stand-alone implementations of the same models. These two models were selected for the current evaluation because we had previously been able to demonstrate nearly exact duplication of results (to within 10^–6^) for stand-alone and template implementation of the models (Bernstein et al., 2023), and because many model features found in other PBPK models for environmental chemicals appear in one or both of these models. In this latter regard, the DCM and CF PBPK models provide good coverage of the model feature space that we sought to address. The results of our experiments have allowed us to draw useful conclusions about which aspects of PBPK model structure, design, and implementation have relatively large (and relatively small) impacts on computational time requirements when performing simulations with such models.

All computational analyses described herein were performed using R version 3.6.0 (R Core Team, 2019) on a Dell Precision T7610 with 32 CPUs and 48 gigabytes of memory running Red Hat Enterprise Linux Workstation release 7.9. Scripts and relevant data files are available online (https://doi.org/10.23719/1531739). The PBPK models used were implemented using the MCSim model specification language (Bois, 2009) and were subsequently translated into C and compiled for use in R. Input parameters and selections of model structure options for specific models are provided via Microsoft Excel files in a pair of input spreadsheets for (1) chemical- and species-specific model parameters and (2) exposure and dosing parameters for specific simulations. Simulations were performed using the “ode” function from the R package “deSolve” (Soetaert et al., 2010) with the “lsoda” ODE integration method. This integration method automatically switches between stiff and non-stiff solution algorithms, and automatically determines the appropriate step-size necessary to maintain the requested error tolerances. Thus, while the user can request simulation results for specific time points, the solver (i.e., the ODE integration algorithm) may use additional time points as necessary to obtain the desired level of numerical accuracy and precision.

For each timing experiment we considered the two different chemical-specific PBPK models (for DCM and CF) and the four different exposure scenarios listed below.

1. Constant continuous inhalation exposure for 2 weeks (assuming a constant concentration of chemical in inhaled air);
2. Constant continuous oral exposure for 2 weeks (assuming a constant, continuous rate of chemical delivery into the stomach compartment);
3. Periodic inhalation exposure (assuming a constant concentration of chemical in inhaled air for 6 hours per day, 5 days per week, for 2 weeks); and
4. Periodic oral exposure (assuming bolus doses were delivered to the stomach compartment six times per day, 7 days per week, for 2 weeks).

For both chemical-specific PBPK models (DCM and CF), we generated ten thousand (10 k) combinations of anatomical and physiological parameters representing a sample of 10 k virtual human subjects using parameter distributions described in the IRIS Toxicological Review of DCM (U.S. EPA, 2011). Then, for each exposure scenario, we ran Monte Carlo simulations using the sample of virtual subjects five times on two different days on our computer (described previously) to assess and account for variations in computational speed. Such variations can arise due to network traffic and background processes that are difficult to control. For the continuous exposure scenarios (1 and 2), the entire sample of 10 k virtual human subjects was used, while for the periodic exposure scenarios (3 and 4) a subset of the sample consisting of 1 k virtual subjects was used because the individual simulations generally required considerably more time in those cases.

The time it took to perform each set of 10 k or 1 k simulations was measured using the “system.time” function in R. We excluded from the timing command those portions of the code that only need to be run once for all 10 k (or 1 k) simulations, and only timed those portions that needed to be run for each of the individual simulations. The timed processes included updating parameter values for each virtual subject in the sample population, setting up “events” data structures (used to implement discrete bolus dosing events), checking for (and computing, if necessary) background rates of exposure, running the model (i.e., computing the solution to the system of differential equations), and returning the list of all simulation output data frames. We also separately timed just the portion of code that runs the model (excluding parameter assignment, creation of data structures, etc.) using the R command “proc.time”. Both the system.time and proc.time commands return the “CPU time” as well as the elapsed (wall clock) time, and in our analysis, we used the CPU time.

In addition to evaluating the differences in computational time when using the PBPK model template vs. stand-alone model implementations, we also evaluated computational time differences when using two approaches for implementing conditional statements used to change model behavior between two options. Such conditional logic may be required for models that allow for simulations of gas uptake from both “open” chambers, in which the concentration is held constant, and “closed” chambers, in which the amount of gas in the chamber decreases over time as it is absorbed and metabolized by the animal(s) in the chamber. One approach is to use model input parameters that are set to either 0 or 1 and then used as multiplicative constants for the relevant mathematical equations or assignment statements in the model, effectively switching them on or off. The other option is to use conditional (“if-then-else”) statements in the model code, which we implemented using the C ternary conditional operator (ISO, 2018). There are 32 conditional statements within the PBPK model template that were implemented with each option: four conditional statements in the “Initialize” section of the model specification file, which are evaluated once per model simulation, and 28 conditional statements in the “Dynamics” section of the model specification file, which are evaluated at each step of the ODE integration algorithm. We hypothesized that use of the conditional operator would require more computational time than use of a multiplicative logical switch parameter.

In some applications, it is important to consider body weight changes over time (e.g., for pregnancy models or simulations of periods that stretch across multiple life stages). To accommodate such applications, the PBPK model template represents body weight as an input variable that can change over time. However, this design choice requires additional unnecessary calculations for scenarios in which body weight is, in fact, constant, and we hypothesized that this may have a significant impact on computational time. To analyze the impact of this design choice, we examined the influence (on simulation time) of allowing body weight (and its dependent parameters) to vary in time by performing simulations with two versions of the PBPK model template: one in which the body weight parameter can vary throughout a simulation and one in which body weight is treated as a constant. Furthermore, for simulations in which body weight was defined as a fixed, constant parameter, we considered subcases in which (1) the dependent parameters were calculated in the “Dynamics” section of the model implementation (and thus calculated at every step of the integration algorithm) and (2) they are calculated only once per simulation in the “Initialize” section. There are 28 parameters in the PBPK model template that depend on body weight that were impacted by these modeling choices.

For some PBPK model compartments and processes, the PBPK model template provides more than one option for representation and implementation based on approaches that are common in published PBPK models. For example, for the venous and arterial blood compartments, one can choose to model the concentration of chemical in both compartments using a steady state approximation described by an algebraic equation. Alternatively, one can choose to avoid the steady state approximation by representing the amount of chemical in each blood compartment using state variables described by differential equations. Similarly, when considering inhalation exposures, one can represent the concentration in the gas exchange region using a steady state approximation for the concentration of chemical in the pulmonary vein. In this case, the modeler can choose to explicitly include a lung tissue compartment; otherwise, and if omitting an explicit lung compartment, the concentration in the pulmonary vein is assumed to transfer directly to the arterial blood compartment. The modeler can also choose to avoid the steady state approximation of the concentration of chemical in the pulmonary vein by representing the amount of substance in the lung with a differential equation that includes terms for the rate of inhalation and exhalation of chemical in the lung compartment. We hypothesized that options that used steady state approximations would require less computational time than those that used additional state variables (i.e., additional differential equations). Note that for the DCM model implementation, using the option that does not include a separate lung compartment also means that lung metabolism is not included, and thus simulations using that version of the model did not include all the biologically relevant features of the original DCM PBPK model.

We considered eight different aspects of PBPK model implementation for our timing experiments. For a given chemical-specific PBPK model (i.e., the DCM or CF model), we compared the computational times required for.

1. Simulations performed using the template implementation of that model and a stand-alone implementation of that model;
2. A template implementation of the model that uses the ternary conditional operator for all conditional statements and a template implementation of the model that uses multiplicative logical switches with value of 1 or 0 for all conditional statements;
3. Simulations using a template implementation of the model with varying numbers of output time points (50, 100, or 500 time points requested) to be returned with the simulation results;
4. Simulations using a template implementation of the model with different numbers of output variables (i.e., calculated quantities that are not state variables) returned with the simulation results (i.e., using 76 or 105 output variables);
5. Simulations using a template implementation of the model when body weight and body weight-dependent quantities are implemented as either being time-varying or fixed (constant) parameters;
6. Simulations using a “full” template implementation of the model that includes equations for all compartments, including compartments that are effectively deactivated because they are not included in the model, and a “reduced” template implementation in which state equations for deactivated compartments were eliminated, reducing the total number of state equations in the model;
7. Simulations using a template implementation of the model that utilizes different (already existing within the model template) options for modeling blood compartments (i.e., using the steady state approximations for the venous and arterial blood compartments vs. representing amounts in these compartments as state variables with separate differential equations for each); and
8. Simulations using a template implementation of the model that utilizes different options for modeling the lung compartment and the gas exchange region (i.e., using the steady state approximation for the gas exchange region vs. representing the amount in this compartment as a state variable, and explicitly including or excluding the lung compartment).

## Results

**FIGURE 3:**
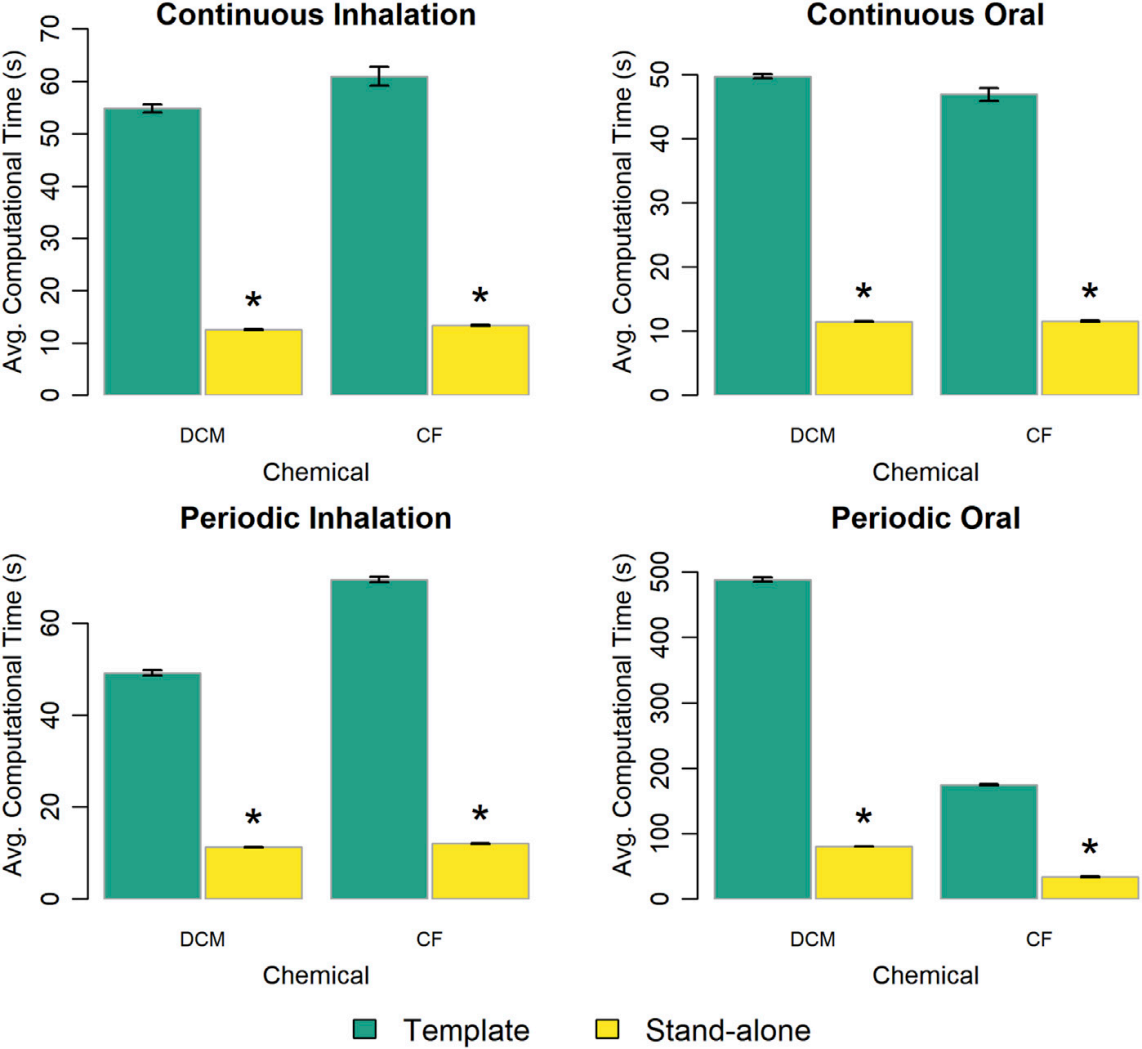
Comparison of the computational time required to run a simulation using the template implementation of a given chemical-specific PBPK model and that required using a stand-alone implementation of that model. The height of each bar represents the mean computational time ( n=10 ) to complete 10 k (for continuous exposures) or 1 k (for periodic exposures) simulations for each model implementation. Error bars show means ± standard deviations in computational time ( n=10 ). Darker and lighter bars represent times required for the PBPK model template implementation and the stand-alone model implementation, respectively, for each chemical. An asterisk above a bar indicates that there was a statistically significant difference ( p<0.05 ) between the average computational times for the template and stand-alone model implementations. A table showing means and standard deviations for computational times for these simulation experiments is provided in the Supplementary Material.

First, we compared the computational times required to perform simulations using a PBPK model template implementation of a chemical-specific PBPK model to that required when using a stand-alone implementation of the same chemical-specific model. Figure 3 summarizes the timing results and shows that the template implementations take 4–6 times longer to run compared to their stand-alone counterparts, depending on the exposure scenario and chemical.

**FIGURE 4:**
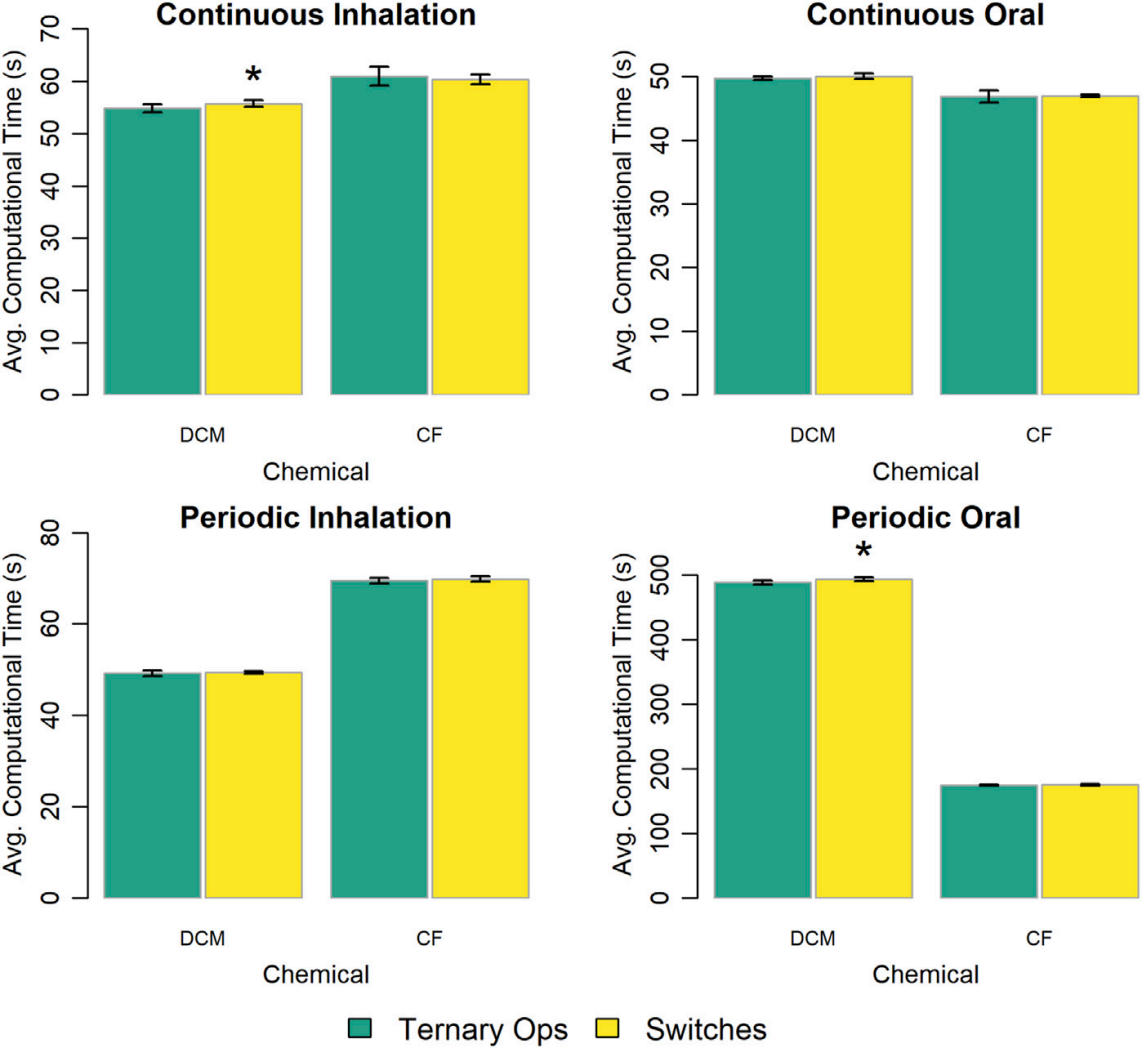
Comparison of the computational times required for simulations with template implementations of the given PBPK model (“DCM” or “CF”) that use different options to implement conditional statements. The height of each bar represents the mean computational time ( n=10 ) to complete 10 k (for continuous exposures) or 1 k (for periodic exposures) simulations for each model implementation. Error bars show means ± standard deviations in computational time ( n=10 ). Darker and lighter bars represent times required for the model implementations using ternary conditional operators (“Ternary Ops”) and multiplicative logical switches (“Switches”), respectively, for all conditional statements. An asterisk above a bar indicates that there was a statistically significant difference ( p<0.05 ) between the average computational times required for the implementations using ternary conditional operators and multiplicative logical switches. A table showing means and standard deviations for computational times for these simulation experiments is provided in the Supplementary Material.

We evaluated two approaches for implementing conditional statements used to change model behavior: the ternary conditional operator (ISO, 2018) and multiplicative logical switches (i.e., Boolean parameters with values of zero or one). Figure 4 summarizes the results of this evaluation. In most cases, the model implementation that used the ternary conditional operator performed slightly faster than the one that used multiplicative logical switches. However, the difference in computational time was less than 2% in all cases.

**FIGURE 5:**
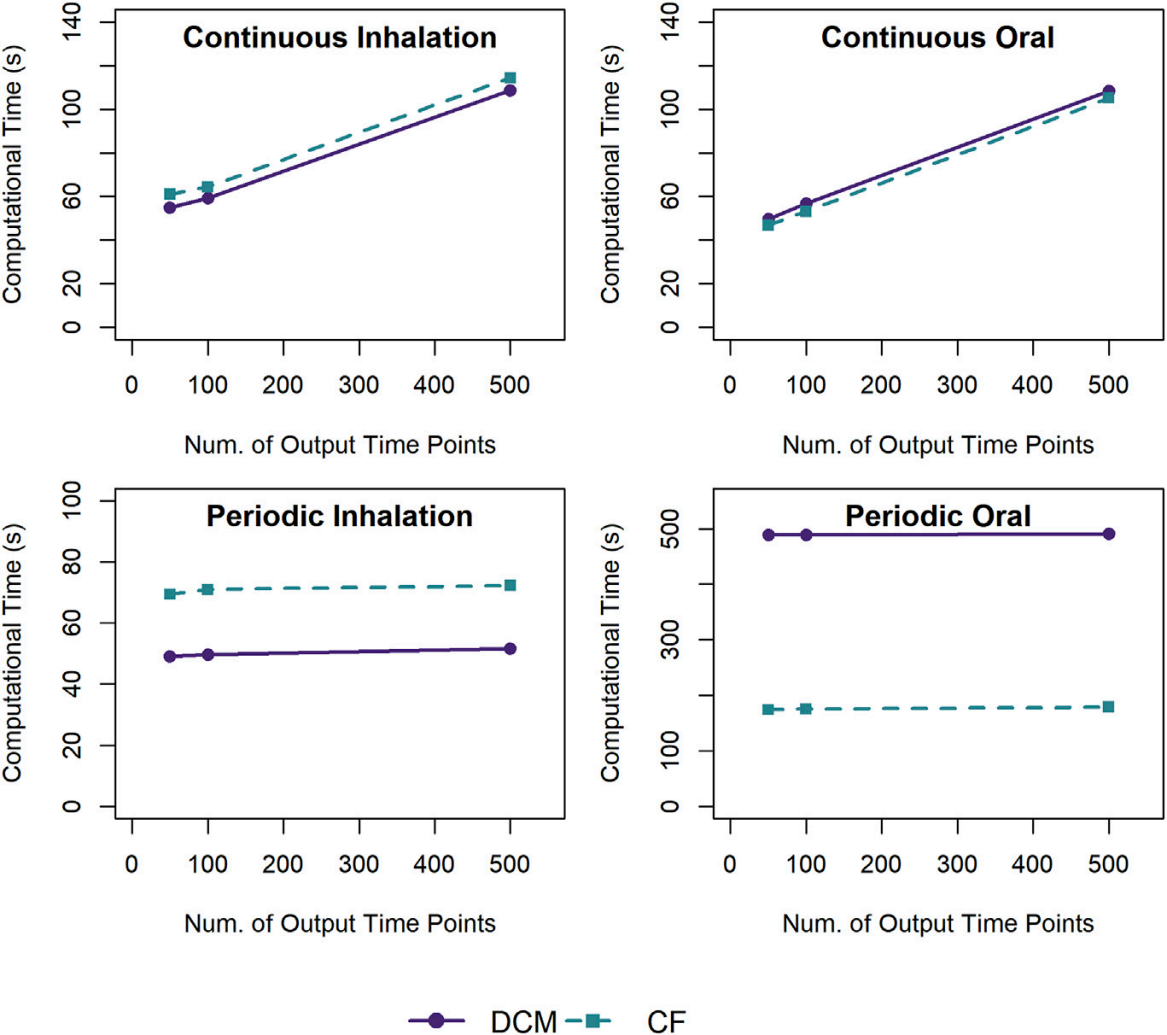
Comparison of the computational times required for simulations using a template implementation of a given PBPK model (“DCM” or “CF”) with different numbers of output time points (50, 100, or 500) to be returned with the simulation results. For each exposure scenario, the solid and dashed lines indicate the results for the average computational times required to perform 10 k (for continuous exposures) or 1 k (for periodic exposures) simulations using the template implementation of the DCM and CF PBPK models, respectively.

We examined how the number of time points at which simulation output values are requested (and returned by the ODE integration algorithm) impacts the computational time, and the results are shown in Figure 5. For the scenarios with constant, continuous exposures, the computational time increases linearly with the number of time points returned. For the scenarios with periodic exposures, the computational time does not vary substantially with the number of time points returned, increasing by less than 1% when increasing the number of requested time points by a factor of 10.

**FIGURE 6:**
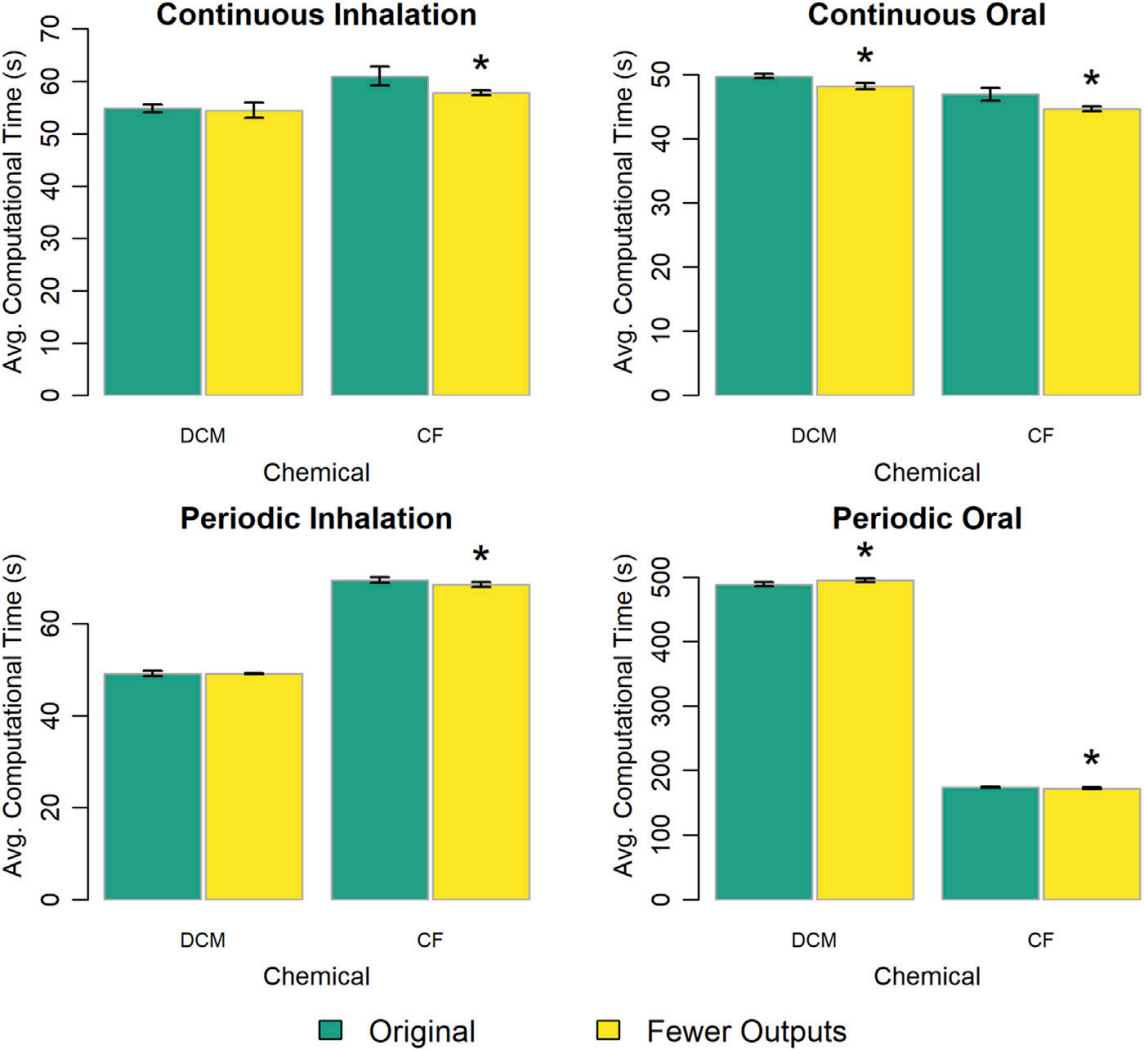
Comparison of the computational times required for simulations using a template implementation of a given PBPK model (“DCM” or “CF”) with different numbers of outputs (i.e., calculated quantities not including state variables) returned with the simulation results. The height of each bar represents the mean computational time ( n=10 ) required to complete 10 k (for continuous exposures) or 1 k (for periodic exposures) simulations for each model implementation. Error bars show means ± standard deviations in computational time ( n=10 ). Darker and lighter bars represent times required using the original PBPK model template (with 105 output variables) and an alternative version of the PBPK model template with fewer (76) output variables. An asterisk above a bar indicates that there was a statistically significant difference ( p<0.05 ) between the average computational times required for simulations using implementations based on the original PBPK model template and the version of the template with fewer output variables. A table showing means and standard deviations for computational times for these simulation experiments is provided in the Supplementary Material.

We created a version of the PBPK model template with approximately 25% fewer output variables to determine the impact of “unused” extra algebraic equations on computational speed. Figure 6 shows that the version of the PBPK model template with fewer output variables typically performed slightly faster than the original version. However, the decrease in computational time was no more than 6% for any given comparison, and in most cases was less than 2%.

**FIGURE 7:**
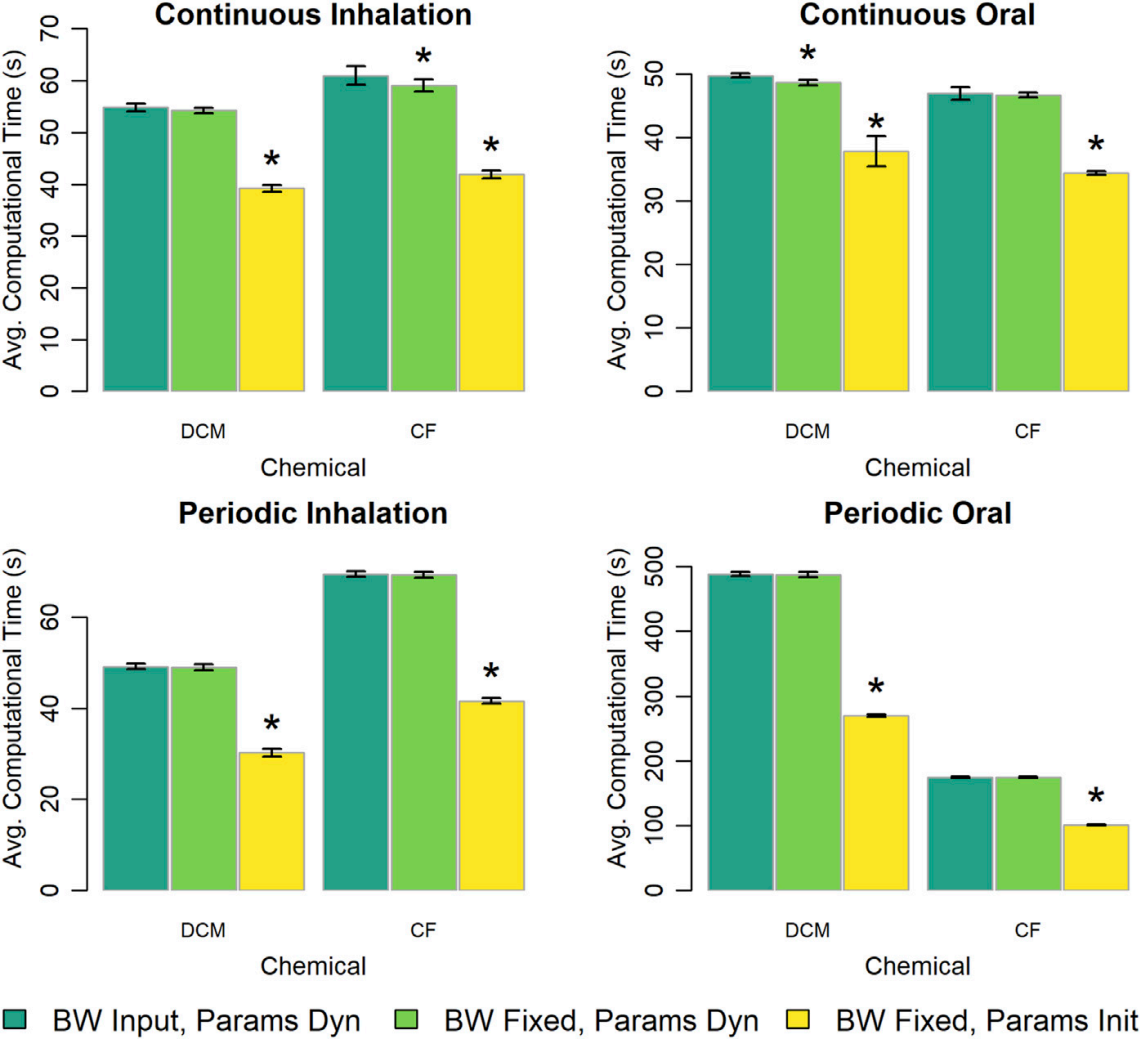
Comparison of the computational times required for simulations using template implementations of a given PBPK model (“DCM” or “CF”) when body weight is treated as time-varying (“BW Input”) or constant (“BW Fixed”) parameters and body weight dependent quantities are treated as possibly dynamically changing throughout the simulation (“Params Dyn”) or are calculated just once during an initialization step (“Params Init”). The height of each bar represents the mean computational time ( n=10 ) required to complete 10 k (for continuous exposures) or 1 k (for periodic exposures) simulations for each model implementation. Error bars show means ± standard deviations in computational time ( n=10 ). In each panel, the darkest bar represents the computational time for the implementation in which body weight is treated as a time-varying input parameter, the middle bar represents the computational time for the implementation in which body weight is treated as a constant parameter but dependent quantities are calculated at each step of the integration algorithm, and the lightest bar represents the computational time for the implementation in which body weight is treated as a constant parameter and dependent quantities are calculated only once per simulation. An asterisk above a bar indicates that there was a statistically significant difference ( p<0.05 ) between the average computational times required when using the implementation in which body weight is treated as a time-varying input parameter and an alternative implementation in which body weight is treated as a constant parameter. Note, for the option in which body weight is treated as a time-varying input parameter, the body weight parameter had a constant value during the entire simulation and was described by a constant-valued input table. A table showing means and standard deviations for computational times for these simulation experiments is provided in the Supplementary Material.

The original version of the PBPK model template treats body weight as a time-varying input parameter, so to evaluate the impact of this design choice on computational time we created a version of the PBPK model template in which body weight is treated as constant. We used the original version of the PBPK model template to perform simulations with a changing body weight and a constant body weight and used the alternative version of the template to perform simulations with a constant body weight. Figure 7 shows the computational time for simulations performed using each of the tested options for describing body weight and its dependent parameters (i.e., those parameters that are calculated based on body weight such as the compartment volumes and blood flow rates). Note that for these results, when body weight is treated as a “constant” for a simulation performed with the original version of the PBPK model template, the body weight parameter is provided as a constant-valued input table. When the body weight dependent parameters are calculated in the “Dynamics” section, the difference in computational time between implementations that use a time-varying body weight input or a fixed body weight input is less than 4%. However, for the alternative version of the PBPK model template, the body weight dependent parameters are calculated only once per simulation (and are therefore also essentially treated as “constants”). Using the alternative “fixed body weight” implementations of the PBPK models there are large savings in computational time with simulations taking up to 30% less time in cases with constant, continuous exposures, and over 40% less time in cases with periodic exposures. We also considered a limited case where the value of the body weight did vary in time and saw that computational time increased compared to the case in which body weight was described by a constant valued function. Details of those results can be found in the Supplementary Material.

**FIGURE 8:**
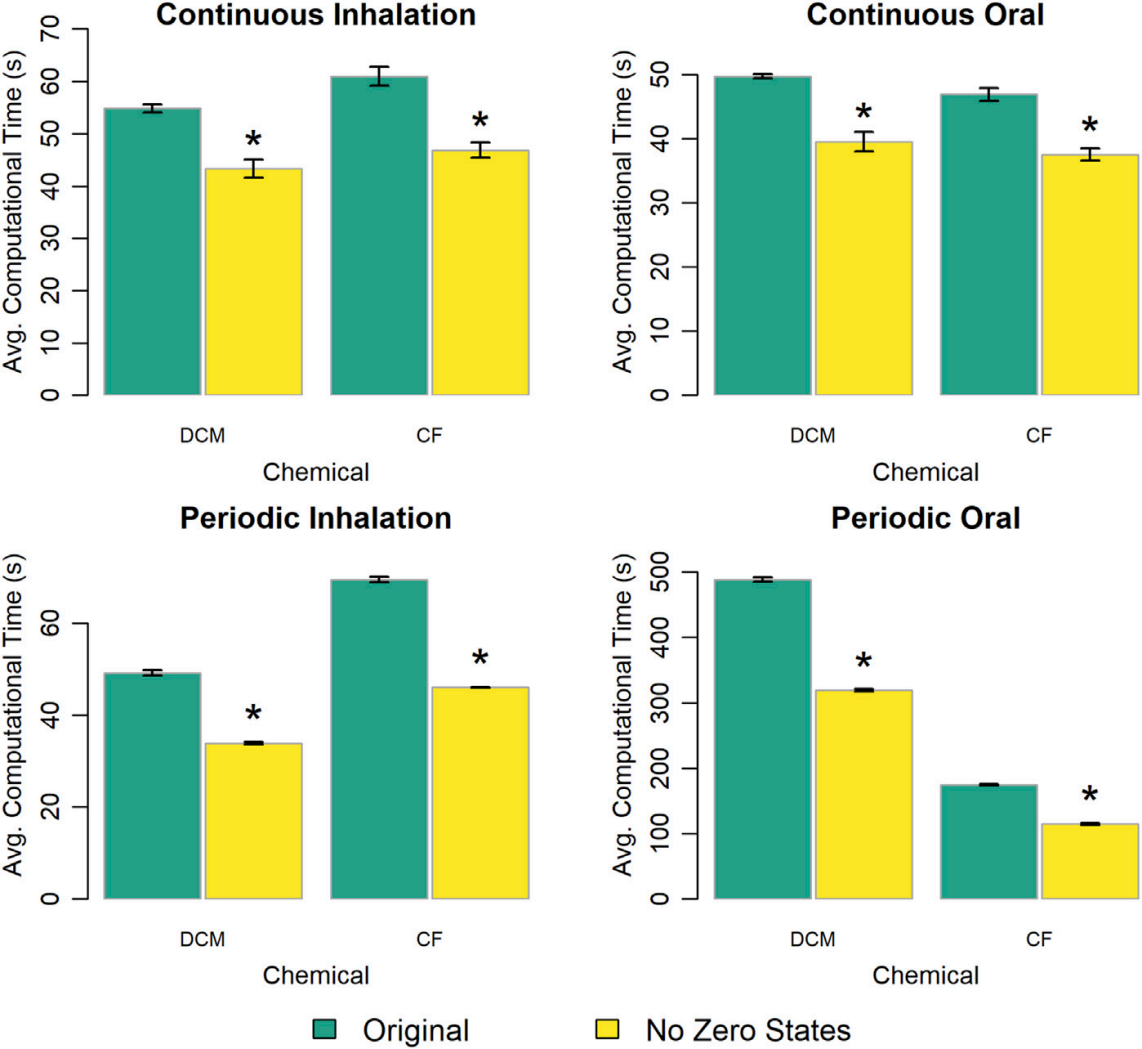
Comparison of the computational times required for simulations using template implementations of a given PBPK model (“DCM” or “CF”) that include (“Original”) or do not include (“No Zero States”) equations for compartments that are deactivated in the full PBPK model template to more efficiently match the chemical-specific PBPK model. The height of each bar represents the mean computational time ( n=10 ) required to complete 10 k (for continuous exposures) or 1 k (for periodic exposures) simulations for each model implementation. Error bars show means ± standard deviations in computational time ( n=10 ). Darker and lighter bars represent times required using the original PBPK model template (with 53 state variables) and an alternative version of the PBPK model template without the deactivated state variables (which had 34 state variables for the DCM model and 33 state variables for the CF model), respectively. An asterisk above a bar indicates that there was a statistically significant difference ( p<0.05 ) between the average computational times required when using the original PBPK model template and the version without the deactivated state variables. A table showing means and standard deviations for computational times for these simulation experiments is provided in the Supplementary Material.

To examine how the inclusion of “unused” state variables, for which time rates of change (i.e., right-hand sides of differential equations) are always equal to zero impacts computational time, we constructed versions of the PBPK model template for each chemical-specific model that removed state variables corresponding to compartments that were deactivated in the full model template implementation. Specifically, we removed 19 of 53 state variables for the modified template implementation of the DCM PBPK model and 20 of 53 state variables for the modified template implementation of the CF PBPK model. Figure 8 shows the computational time results for this experiment. Removing the unused state variables resulted in simulation times 20%–35% lower than times for simulations performed with the full PBPK model template.

**FIGURE 9:**
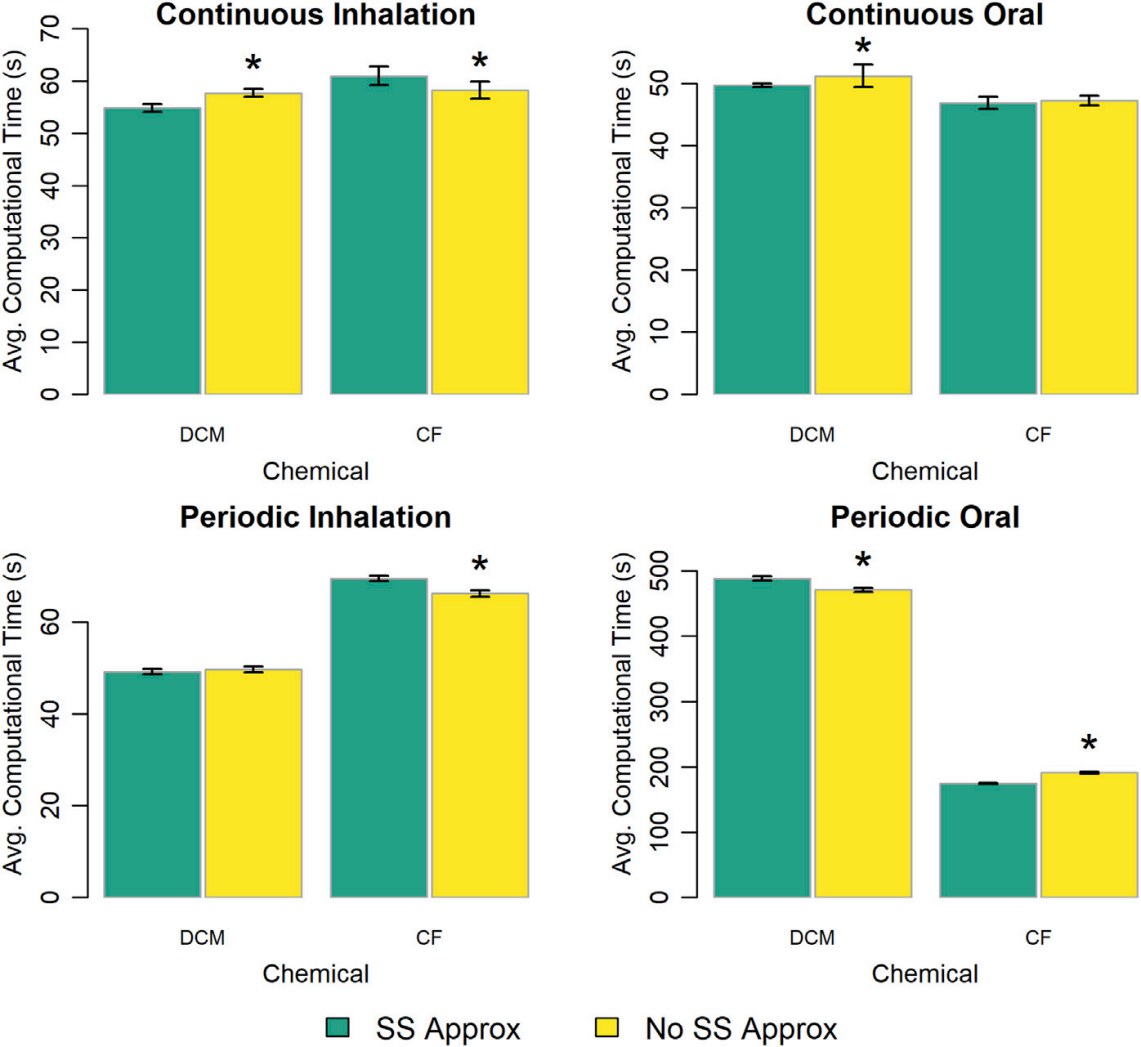
Comparison of the computational times required for simulations using template implementations of a given PBPK model (“DCM” or “CF”) that utilize different (already existing within the model template) options (“SS Approx” or “No SS Approx”) for modeling blood compartments. The heigh of each bar represents the mean computational time ( n=10 ) required to complete 10 k (for continuous exposures) or 1 k (for periodic exposures) simulations for each model implementation. Error bars show means ± standard deviations in computational time ( n=10 ). Darker and lighter bars represent times required when using steady state approximations or state variables, respectively, to represent the concentrations (or amounts) of chemical in the venous and arterial blood. An asterisk above a bar indicates that there was a statistically significant difference ( p<0.05 ) between the average computational times required when using steady state approximations or state variables to represent blood compartment concentrations (or amounts). A table showing means and standard deviations for computational times for these simulation experiments is provided in the Supplementary Material.

The PBPK model template provides two options for estimating the amount of chemical in the venous and arterial blood compartments: (1) using steady state approximations to model the concentration of chemical in each blood compartment or (2) representing the amounts of chemical in those compartments with state variables described by differential equations. Figure 9 shows the computational time required for each exposure scenario using each modeling option. There is no clear pattern as to which modeling option results in faster simulations, and in most of the cases we considered the differences in computational times were less than 5%.

**FIGURE 10:**
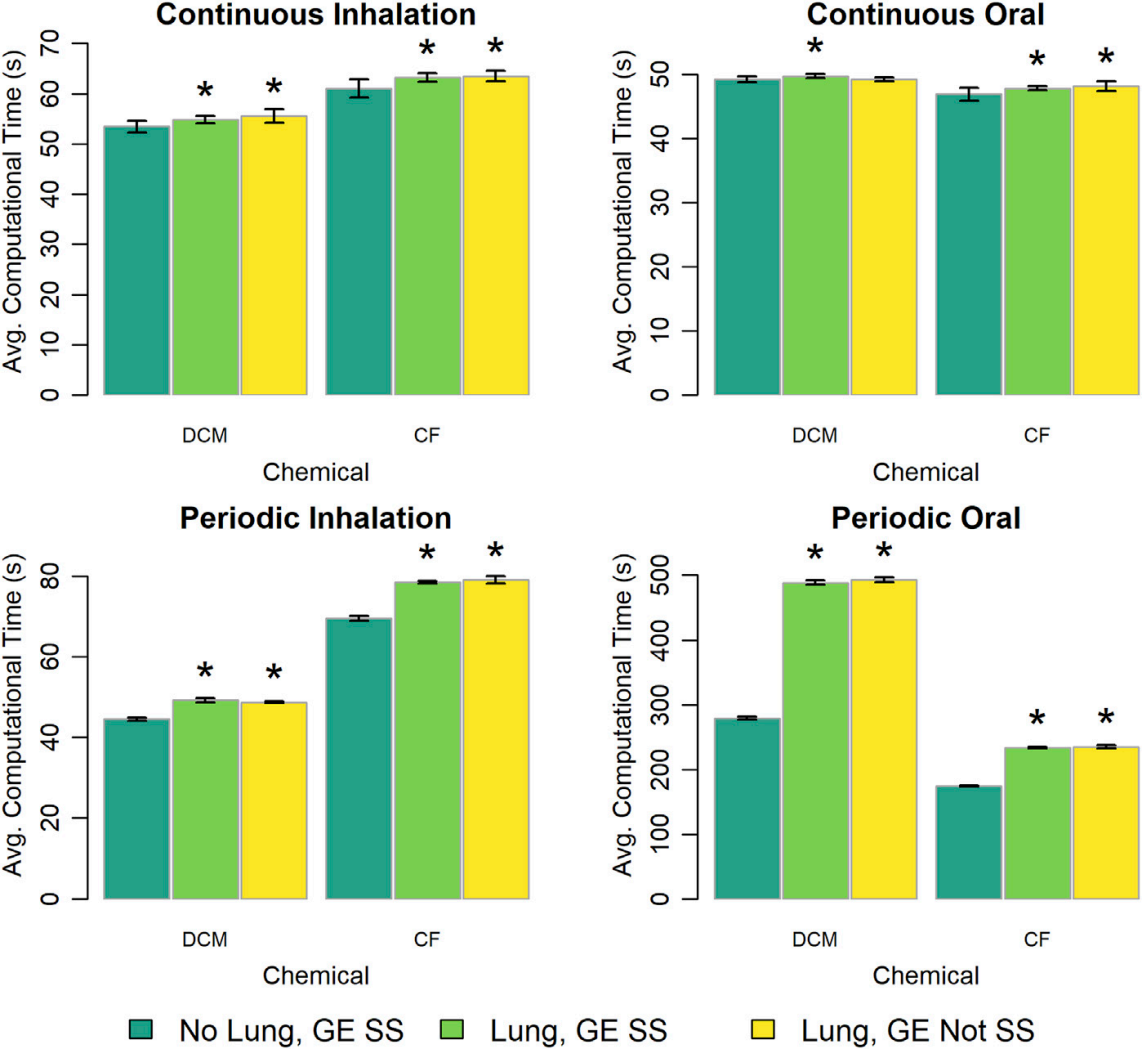
Comparison of the computational times required for simulations using template implementations of a given (“DCM” or “CF”) model that utilize different options (“No Lung” vs. “Lung” and “GE SS” vs. “GE Not SS”) for modeling the lung compartment and the gas exchange region. The heigh of each bar represents the mean computational time ( n=10 ) required to complete 10 k (for continuous exposures) or 1 k (for periodic exposures) simulations for each model implementation. Error bars show means ± standard deviations in computational time ( n=10 ). In each panel, the darkest bar represents the computational time for the implementation with no explicit lung compartment and a steady state approximation of the concentration of chemical in the gas exchange region, the middle bar represents the computational time for the implementation with an explicit lung compartment and a steady state approximation of the concentration of chemical in the gas exchange region, and the lightest bar represents the computational time for the implementation with an explicit lung compartment with terms describing the rates of inhalation and exhalation of chemical. An asterisk above a bar indicates that there was a statistically significant difference ( p<0.05 ) between the average computational times required when using the implementations with or without an explicit lung compartment. Note that for DCM, when using the implementation without an explicit lung compartment, lung metabolism was excluded from the model (even though such metabolism is an inherent feature of the original DCM PBPK model). A table showing means and standard deviations for computational times for these simulation experiments is provided in the Supplementary Material.

The PBPK model template provides three options for representing the lung compartment and gas exchange region. We explored differences in computational time requirements for each of these options and the results are shown in Figure 10. For the constant, continuous exposure scenarios there was less than a 5% difference in the computational times for each of the three modeling options. However, for the periodic exposure scenarios there were significant differences (p < 10^–13^) between the computational times for the options that included an explicit lung compartment and the computational times for the option that did not. Including an explicit lung compartment increased simulation time by about 10% for periodic inhalation exposures, while for periodic oral exposures simulation time increased by 75% with the DCM model implementation and by 35% with the CF model implementation.

## Discussion

Based on our experiments, simulations using template-implemented models take longer than simulations using equivalent stand-alone models. This is expected because the PBPK model template includes 53 state variables (quantities described by differential equations) and 105 output variables (quantities described by algebraic equations), while the stand-alone implementation of the DCM model includes 21 state variables and 22 output variables, and the stand-alone implementation of the CF model includes 19 state variables and 26 output variables. As we demonstrated in our evaluation of the impact of removing state variables for which rates of change are always equal to zero (see Figure 8), the inclusion of additional state variables (and corresponding differential equations) in the model can have a large impact on the computational time needed for simulations, and the PBPK model template has more than double the number of state variables of either of the two stand-alone model implementations we considered. However, including these additional state variables gives the model template more flexibility and enables implementation of a greater variety of chemical-specific PBPK models. While the DCM and CF models described herein contain similar numbers of state variables in their stand-alone implementations, when mapped to the model template superstructure, different state variables were chosen in order to implement each model (see Figures 1, 2).

Representation of body weight as either a constant or as a (possibly) time-varying input parameter also influenced computational time. In particular, defining body weight as a constant (fixed) parameter and calculating all dependent quantities once per simulation led to a large time savings compared to an approach in which body weight dependent quantities were calculated at each time step of the integration algorithm. For both of the chemical-specific PBPK models we considered, the stand-alone model implementations represented body weight as a constant parameter, and thus utilized the fastest of the three options for representing body weight (and related quantities) that we considered. However, if one wanted to use either of those models to perform simulations in which body weight changes might be important (e.g., simulations of periods of time during which the person or laboratory animal was growing), those implementations would be insufficient. For such a simulation, one would need to create a new stand-alone model implementation that represents body weight and its dependent quantities as time-varying quantities. In this case, one would need to implement the model and perform QA review, which could require additional time and effort. Because body weight is represented in the PBPK model template as an input parameter (that can have different values at different times in the simulation), it can be used for scenarios where body weight varies over time and scenarios where body weight is constant over time (by providing a constant valued input table) without needing to change the underlying model file. Therefore, a user does not need to spend additional time implementing and performing QA review of a new model version that will accommodate time-varying body weight. However, converting a model that includes changing body weight to one where body weight is assumed to be fixed would require additional effort, so providing a version of the model template that has a fixed body weight may be advantageous for PBPK modeling scenarios in which body weight is assumed to be constant.

For constant, continuous exposure scenarios, the number of output simulation time points requested significantly impacted computational time. The differences between results for constant and periodic exposure inputs are not surprising since periodic exposures involve regular, recurring discontinuities in some of the state variables throughout a simulation and thus the step-size used by the solver to attain the desired error tolerance is likely smaller than that requested by the user for output. On the other hand, for constant, continuous exposures, the system reaches a steady state, and the solver can use much larger time steps while maintaining the desired error tolerance. Thus, for the constant and continuous exposure cases, requesting a larger number of output time points requires the solver to evaluate the system at additional time points in order to include those values in the output. Modelers performing many simulations should carefully consider how many time points will be needed for their subsequent analyses. Requesting more time points than the solver requires to attain the desired numerical accuracy can greatly increase the computational time, especially for constant, continuous exposure scenarios.

Some model implementation choices had little impact on simulation speed; that is, the difference in the computational time with each option was small relative to the average time required (for either option). For example, using different approaches for implementing conditional statements impacted computational time by less than 2% and implementing blood compartments in different ways impacted computational time by less than 5%. Including 25% fewer output variables yielded a less than 6% decrease in computational time, implying that the convenience of having access to any outputs that may be of interest to modelers may justifies their inclusion in the PBPK model template.

Selecting between various options for representing gas exchange in a PBPK model also had some impact on computational time, but the largest time differences were observed between simulations involving implementations that did or did not include an explicit lung compartment rather than implementations that did or did not use steady-state approximations for concentrations in specific regions of the body. This makes sense because including an explicit lung compartment requires that the model include an additional state variable (with a non-zero rate of change), and we demonstrated in separate simulation experiments (described herein) that the inclusion of additional state variables has a significant impact on the computational speed of simulations. However, using a steady state approximation or a differential equation to represent the concentration (or amount) of substance in the gas exchange region had only a small impact on computational time. This finding was comparable to the result observed for simulation experiments involving a similar representation choice for the blood compartments.

While implementations using the PBPK model template take more computational time than simulations using dedicated models, there are still advantages to using the model template. The template allows for faster and more efficient QA review, which typically includes (among other things) checking that the model implementation is accurate. Generally, this portion of the QA review can require many hours of work, but when using a model template implementation, much of this portion of the QA review can be considered complete [because QA review of the PBPK model template has been performed by the authors of the PBPK model template (Bernstein et al., 2021; Bernstein et al., 2023)]. Only the input spreadsheets describing the parameters for the model and exposure scenarios need to be checked. Similarly, when implementing a model, either *de novo* or from a published source, using the PBPK model template is faster than developing a stand-alone model implementation since only the input spreadsheets need to be completed. For *de novo* models this also allows for greater consistency in implementation of specific features since all template-implemented models will use the same form for the equations.

When one plans to use a PBPK model to perform Monte Carlo simulations or do other analyses requiring many hundreds or thousands of simulations, parallel processing should be considered. Since simulations for each member of a virtual population are independent, they can be performed at the same time using different processor cores. Parallel processing allows one to take advantage of the multiple cores (or “workers”) available on most modern computers to complete multiple independent simulations more quickly. For the analysis described here, none of the model simulations were performed in parallel, but doing so can decrease computational time considerably. One can take advantage of parallelization to perform multiple independent simulations more quickly using any PBPK model implementation. So, if performing a simulation requires 1 s using implementation A and 5 s using implementation B, then if one performs thousands of simulations using parrallel computing with implementation A, one can expect that the complete set of simulations will be performed in about 20% of the time required when using parallel computing with implementation B. Because the time savings inherent in the use of parallel processing applies to all model implementations equally, we did not explore it here.

In performing this work, we also found that computational speed was sometimes impacted by factors outside of our control related to the specific computers, environments, and systems we were using. We initially used a standard-issue corporate laptop (a Dell Latitude 5300 two-in-1 computer running Microsoft Windows 10) and the time to simulate 10 k virtual individuals exposed via inhalation to a constant air concentration of DCM ranged from 36.72 to 68.44 s (with relatively high variability), even after ensuring that the laptop was disconnected from the network and stopping execution of all unnecessary programs we could control. In comparison, when we switched to using the Dell Precision T7610 running Red Hat Enterprise Linux Workstation release 7.9 (which was used for all results provided herein), the time to perform those same simulations ranged from 53.79 to 59.53 s (with relatively low variability). Such variability in computational time can be difficult to control, and it may make it more difficult to determine the significance of small differences in computational time, such as that as seen in Figures 4, 9, in which some of the differences appear to be statistically significant ( p<0.05 ), but there is no clear pattern in which of the tested options results in lesser computational times.

For the research presented here, we considered the impact of different modeling choices on computational speed, but we did not consider the impact of these choices on model predictions. In particular, we did not consider how the various options and assumptions used to describe the blood compartments and representation of inhalation-relevant aspects of a PBPK model might impact predictions of time-course concentrations. We plan to explore this in future work.

## Conclusion

How one chooses to implement a PBPK model can have a significant impact on computational time required for simulations. The time required for a single simulation can be especially important for Monte Carlo PBPK modeling analyses, which can involve many thousands of simulations. We performed PBPK model simulation timing experiments considering various aspects of model design implementation and showed which design and implementation choices have greater and lesser impacts on computational time. We also demonstrated that using our PBPK model template to implement a chemical-specific model can result in longer simulation times compared to those that may be achieved with a stand-alone implementation of the same PBPK model. However, we identified several of the factors that contribute to the greater computational time requirements for PBPK model template implementations of models (such as a relatively large number of compartments represented by state variables and a time-varying input variable to represent body weight) and determined that these factors are also beneficial features of the PBPK model template that give it flexibility and broader applicability than the individual stand-alone model implementations. This flexibility, as well as the (non-computational, human) time savings realized when implementing and reviewing a model using the PBPK model template, may offset or justify the additional computational time required for PBPK model template simulations in many cases. Whether one uses the PBPK model template or some other implementation strategy, our results provide insights into some of the features and design choices that determine the computational expense of PBPK model simulations and may therefore prove useful to anyone seeking to develop, improve, or apply a PBPK model.

## Data Availability

The datasets presented in this study can be found in online repositories. The names of the repository/repositories and accession number(s) can be found in the article/Supplementary Material.
