# Supplementary material for: Comparing computational times for simulations when using PBPK model template and stand-alone implementations of PBPK models
Source: Front Toxicol. 2025 Feb 19;7:1518769. doi: 10.3389/ftox.2025.1518769 (PMC11880222; doi:10.3389/ftox.2025.1518769)
Supplement: Supplementary file 1 [file Table1.docx]

**Supplemental Material**

Summary Tables for Computational Timing Experiments

These tables summarize the information shown in Figures 3, 4, 6-10.

Table 1 Comparison of the computational time required for simulations using the template implementation of a given chemical-specific PBPK model (“DCM” or “CF”) and a stand-alone implementation of that model.

| Exposure Scenario | | Time to Perform Simulations (s) | | | |
| --- | --- | --- | --- | --- | --- |
|  | | DCM | | CF | |
|  |  | Model Template Implementation | Stand Alone Model | Model Template Implementation | Stand Alone Model |
| Constant Inhalation  (10k simulations) | Avg. | 54.83 | 12.49 | 61.00 | 13.33 |
|  | St. Dev. | 0.7367 | 0.1296 | 1.823 | 0.1576 |
|  | Range | 53.99 – 56.41 | 12.38 – 12.76 | 58.54 – 63.95 | 13.20 – 13.72 |
| Constant Oral  (10k simulations) | Avg. | 49.72 | 11.48 | 46.88 | 11.49 |
|  | St. Dev. | 0.3196 | 0.04403 | 0.9765 | 0.09091 |
|  | Range | 49.20 – 50.27 | 11.42 – 11.55 | 45.81 – 48.90 | 11.40 – 11.68 |
| Periodic Inhalation  (1k simulations) | Avg. | 49.20 | 11.18 | 69.50 | 11.99 |
|  | St. Dev. | 0.5708 | 0.07377 | 0.5791 | 0.1013 |
|  | Range | 48.54 – 50.26 | 11.09 – 11.31 | 68.84 – 70.48 | 11.86 – 12.12 |
| Periodic Oral  (1k simulations) | Avg. | 488.5 | 79.90 | 174.0 | 33.40 |
|  | St. Dev. | 3.171 | 0.3067 | 1.027 | 1.096 |
|  | Range | 485.0 – 492.8 | 79.54 – 80.40 | 172.4 – 175.4 | 32.51 – 35.53 |

Table 2 Comparison of the computational time required for simulations using a template implementation of a given chemical-specific PBPK model (“DCM” or “CF”) that uses the ternary operator for all conditional statements to the time required for a template implementation of the same model that uses switches with value of 1 or 0 for all conditional statements.

| Exposure Scenario | | Time to Perform Simulations (s) | | | |
| --- | --- | --- | --- | --- | --- |
|  | | DCM | | CF | |
|  |  | Using Ternary Conditional Operators | Using [0,1] Valued Switches | Using Ternary Conditional Operators | Using [0,1] Valued Switches |
| Constant Inhalation  (10k simulations) | Avg. | 54.83 | 55.70 | 61.00 | 60.36 |
|  | St. Dev. | 0.7367 | 0.6356 | 1.823 | 0.9080 |
|  | Range | 53.99 – 56.41 | 55.02 – 56.80 | 58.54 – 63.95 | 58.92 – 61.36 |
| Constant Oral  (10k simulations) | Avg. | 49.72 | 50.06 | 46.88 | 46.98 |
|  | St. Dev. | 0.3196 | 0.4355 | 0.9765 | 0.1954 |
|  | Range | 49.20 – 50.27 | 49.51 – 50.98 | 45.81 – 48.90 | 46.62 – 47.26 |
| Periodic Inhalation  (1k simulations) | Avg. | 49.20 | 49.4 | 69.50 | 69.83 |
|  | St. Dev. | 0.5708 | 0.2732 | 0.5791 | 0.6196 |
|  | Range | 48.54 – 50.26 | 48.97 – 49.75 | 68.84 – 70.48 | 69.01 – 70.75 |
| Periodic Oral  (1k simulations) | Avg. | 488.5 | 493.5 | 174.0 | 174.9 |
|  | St. Dev. | 3.171 | 2.801 | 1.027 | 1.348 |
|  | Range | 485.0 – 492.8 | 490.5 – 498.2 | 172.4 – 175.4 | 173.0 – 176.9 |

Table 3 Comparison of the computational time required for simulations using a template implementation of a given chemical-specific PBPK model (“DCM” or “CF”) with different numbers of outputs (i.e., calculated quantities not including state variables) returned with the simulation results.

| Exposure Scenario | | Time to Perform Simulations (s) | | | |
| --- | --- | --- | --- | --- | --- |
|  | | DCM | | CF | |
|  |  | Using Original Template Implementation (105 Outputs) | Returning Fewer Outputs (76 Outputs) | Using Original Template Implementation (105 Outputs) | Returning Fewer Outputs (76 Outputs) |
| Constant Inhalation  (10k simulations) | Avg. | 54.83 | 54.45 | 61.00 | 57.81 |
|  | St. Dev. | 0.7367 | 1.449 | 1.823 | 0.4700 |
|  | Range | 53.99 – 56.41 | 53.11 – 57.00 | 58.54 – 63.95 | 57.28 – 58.90 |
| Constant Oral  (10k simulations) | Avg. | 49.72 | 48.19 | 46.88 | 44.67 |
|  | St. Dev. | 0.3196 | 0.4902 | 0.9765 | 0.3704 |
|  | Range | 49.20 – 50.27 | 47.57 – 48.92 | 45.81 – 48.90 | 44.13 – 45.25 |
| Periodic Inhalation  (1k simulations) | Avg. | 49.20 | 49.13 | 69.50 | 68.59 |
|  | St. Dev. | 0.5708 | 0.1465 | 0.5791 | 0.5473 |
|  | Range | 48.54 – 50.26 | 48.87 – 49.45 | 68.84 – 70.48 | 68.01 – 69.34 |
| Periodic Oral  (1k simulations) | Avg. | 488.5 | 495.0 | 174.0 | 172.3 |
|  | St. Dev. | 3.171 | 2.735 | 1.027 | 1.5767 |
|  | Range | 485.0 – 492.8 | 491.1 – 499.2 | 172.4 – 175.4 | 170.8 – 174.3 |

Table 4 Comparison of the computational time required for simulations using a template implementation of a given chemical-specific PBPK model (“DCM” or “CF”) when body weight (“BW”) and body weight-dependent quantities are implemented as either being time-varying or fixed parameters.

| Exposure Scenario | | Time to Perform Simulations (s) | | |
| --- | --- | --- | --- | --- |
|  | | BW as Input Parameter* | BW as Fixed Parameter, Dependent Parameters in Dynamics Section | BW as Fixed Parameter, Dependent Parameters in Initialize Section |
|  |  | DCM | | |
| Constant Inhalation  (10k simulations) | Avg. | 54.83 | 54.28 | 39.20 |
|  | St. Dev. | 0.7367 | 0.5359 | 0.6618 |
|  | Range | 53.99 – 56.41 | 53.25 – 54.97 | 38.44 – 40.65 |
| Constant Oral  (10k simulations) | Avg. | 49.72 | 48.58 | 37.78 |
|  | St. Dev. | 0.3196 | 0.4236 | 2.356 |
|  | Range | 49.20 – 50.27 | 48.16 – 49.50 | 35.53 – 41.83 |
| Periodic Inhalation  (1k simulations) | Avg. | 49.20 | 49.03 | 30.20 |
|  | St. Dev. | 0.5708 | 0.6523 | 0.8134 |
|  | Range | 48.54 – 50.26 | 48.38 – 50.51 | 29.21 – 31.53 |
| Periodic Oral  (1k simulations) | Avg. | 488.5 | 487.7 | 269.5 |
|  | St. Dev. | 3.171 | 4.103 | 1.762 |
|  | Range | 485.0 – 492.8 | 483.2 – 494.2 | 267.8 – 272.7 |
|  | | CF | | |
| Constant Inhalation  (10k simulations) | Avg. | 61.00 | 59.05 | 41.88 |
|  | St. Dev. | 1.823 | 1.153 | 0.7310 |
|  | Range | 58.54 – 63.95 | 57.73 – 61.48 | 41.32 – 43.43 |
| Constant Oral  (10k simulations) | Avg. | 46.88 | 46.64 | 34.37 |
|  | St. Dev. | 0.9765 | 0.3846 | 0.2647 |
|  | Range | 45.81 – 48.90 | 46.21 – 47.29 | 33.96 – 34.86 |
| Periodic Inhalation  (1k simulations) | Avg. | 69.50 | 69.31 | 41.62 |
|  | St. Dev. | 0.5791 | 0.6579 | 0.5614 |
|  | Range | 68.84 – 70.48 | 68.59 – 70.74 | 41.14 – 42.66 |
| Periodic Oral  (1k simulations) | Avg. | 174.0 | 174.3 | 100.2 |
|  | St. Dev. | 1.027 | 0.6812 | 0.9547 |
|  | Range | 172.4 – 175.4 | 173.6 – 175.6 | 99.32 – 101.5 |

*Note, BW is constant valued over the length of the simulation and described by a constant valued input table.

Table 5 Comparison of the computational time required for simulations using a template implementation of a given chemical-specific PBPK model (“DCM” or “CF”) that includes (or does not include) equations for compartments that are deactivated in the full PBPK model template to match the chemical-specific PBPK model.

| Exposure Scenario | | Time to Perform Simulations (s) | | | |
| --- | --- | --- | --- | --- | --- |
|  | | DCM | | CF | |
|  |  | Using Original Template Implementation (53 States) | Removing “Zero-Valued” States (34 States) | Using Original Template Implementation (53 States) | Removing “Zero-Valued” States (33 States) |
| Constant Inhalation  (10k simulations) | Avg. | 54.83 | 43.32 | 61.00 | 46.88 |
|  | St. Dev. | 0.7367 | 1.745 | 1.823 | 1.482 |
|  | Range | 53.99 – 56.41 | 41.94 – 47.68 | 58.54 – 63.95 | 45.17 – 48.63 |
| Constant Oral  (10k simulations) | Avg. | 49.72 | 39.53 | 46.88 | 37.51 |
|  | St. Dev. | 0.3196 | 1.516 | 0.9765 | 0.9876 |
|  | Range | 49.20 – 50.27 | 38.58 – 42.51 | 45.81 – 48.90 | 36.47 – 39.69 |
| Periodic Inhalation  (1k simulations) | Avg. | 49.20 | 33.90 | 69.50 | 46.01 |
|  | St. Dev. | 0.5708 | 0.2514 | 0.5791 | 0.1306 |
|  | Range | 48.54 – 50.26 | 33.57 – 34.24 | 68.84 – 70.48 | 45.85 – 46.29 |
| Periodic Oral  (1k simulations) | Avg. | 488.5 | 319.4 | 174.0 | 114.1 |
|  | St. Dev. | 3.171 | 1.856 | 1.027 | 1.784 |
|  | Range | 485.0 – 492.8 | 317.3 – 323.1 | 172.4 – 175.4 | 112.7 – 117.8 |

Table 6 Comparison of the computational time required for simulations using a template implementation of a given chemical-specific PBPK model (“DCM” or “CF”) that utilizes different options (available in the PBPK model template) for representing blood compartments.

| Exposure Scenario | | Time to Perform Simulations (s) | | | |
| --- | --- | --- | --- | --- | --- |
|  | | DCM | | CF | |
|  |  | Using Steady State Approx. for Blood Comp. | Not Using Steady State Approx. for Blood Comp. | Using Steady State Approx. for Blood Comp. | Not Using Steady State Approx. for Blood Comp. |
| Constant Inhalation  (10k simulations) | Avg. | 54.83 | 57.71 | 61.00 | 58.23 |
|  | St. Dev. | 0.7367 | 0.7309 | 1.823 | 1.632 |
|  | Range | 53.99 – 56.41 | 56.76 – 59.23 | 58.54 – 63.95 | 56.68 – 61.38 |
| Constant Oral  (10k simulations) | Avg. | 49.72 | 51.26 | 46.88 | 47.29 |
|  | St. Dev. | 0.3196 | 1.826 | 0.9765 | 0.1835 |
|  | Range | 49.20 – 50.27 | 49.74 – 54.78 | 45.81 – 48.90 | 46.63 – 49.31 |
| Periodic Inhalation  (1k simulations) | Avg. | 49.20 | 49.66 | 69.50 | 66.22 |
|  | St. Dev. | 0.5708 | 0.6135 | 0.5791 | 0.7340 |
|  | Range | 48.54 – 50.26 | 49.05 – 50.68 | 68.84 – 70.48 | 64.97 – 67.19 |
| Periodic Oral  (1k simulations) | Avg. | 488.5 | 471.3 | 174.0 | 190.8 |
|  | St. Dev. | 3.171 | 3.404 | 1.027 | 1.163 |
|  | Range | 485.0 – 492.8 | 468.5 – 478.2 | 172.4 – 175.4 | 189.4 – 192.5 |

Table 7 Comparison of the computational time required for simulations using a template implementation of a given chemical-specific PBPK model (“DCM” or “CF”) that utilizes different options (available in the PBPK model template) for representing the lung compartment and the gas exchange region.

| Exposure Scenario | | Time to Perform Simulations (s) | | |
| --- | --- | --- | --- | --- |
|  | | No Explicit Lung Comp., Using Steady State Approx. for Gas Exchange Comp.* | Explicit Lung Comp., Using Steady State Approx. for Gas Exchange Comp. | Explicit Lung Comp., Not Using Steady State Approx. for Gas Exchange Comp. |
|  |  | DCM | | |
| Constant Inhalation  (10k simulations) | Avg. | 53.47 | 54.83 | 55.57 |
|  | St. Dev. | 1.109 | 0.7367 | 1.318 |
|  | Range | 52.10 – 55.28 | 53.99 – 56.41 | 54.17 – 58.79 |
| Constant Oral  (10k simulations) | Avg. | 49.19 | 49.72 | 49.20 |
|  | St. Dev. | 0.4371 | 0.3196 | 0.3118 |
|  | Range | 48.67 – 49.98 | 49.20 – 50.27 | 48.72 – 49.85 |
| Periodic Inhalation  (1k simulations) | Avg. | 44.54 | 49.20 | 48.71 |
|  | St. Dev. | 0.3610 | 0.5708 | 0.1855 |
|  | Range | 44.00 – 44.98 | 48.54 – 50.26 | 48.44 – 48.98 |
| Periodic Oral  (1k simulations) | Avg. | 279.2 | 488.5 | 493.1 |
|  | St. Dev. | 2.093 | 3.171 | 3.516 |
|  | Range | 275.5 – 281.9 | 485.0 – 492.8 | 489.6 – 500.3 |
|  | | CF | | |
| Constant Inhalation  (10k simulations) | Avg. | 61.00 | 63.22 | 63.54 |
|  | St. Dev. | 1.823 | 0.8574 | 0.5384 |
|  | Range | 58.54 – 63.95 | 62.02 – 64.86 | 62.56 – 65.31 |
| Constant Oral  (10k simulations) | Avg. | 46.88 | 47.79 | 48.15 |
|  | St. Dev. | 0.9765 | 0.3315 | 0.7463 |
|  | Range | 45.81 – 48.90 | 47.19 – 48.33 | 47.46 – 49.68 |
| Periodic Inhalation  (1k simulations) | Avg. | 69.50 | 78.43 | 79.07 |
|  | St. Dev. | 0.5791 | 0.2846 | 0.9483 |
|  | Range | 68.84 – 70.48 | 78.04 – 79.06 | 78.01 – 80.42 |
| Periodic Oral  (1k simulations) | Avg. | 174.0 | 233.6 | 234.9 |
|  | St. Dev. | 1.027 | 0.9619 | 2.252 |
|  | Range | 172.4 – 175.4 | 232.6 – 235.2 | 232.7 – 239.0 |

*When using this modeling option for the DCM model, lung metabolism must be excluded, while it is included for the other two options.

Impact of Changing Body Weight on Computational Time

To test if using a body weight described by a function that changes in time affects computational time, we tested a limited case. We described body weight using an input table with body weights provided at three times: the start, midpoint, and end of the simulation. Body weight was then given as

Body Weight = [BW_initial, Factor*BW_initial, BW_Initial].

We used 4 values for the Factor variable: [1, 1.2, 1.5, 2]. We then performed the experiment for each chemical and exposure scenario as we did for the other tested experiments. The plots below show the impact on computational time for each exposure scenario.


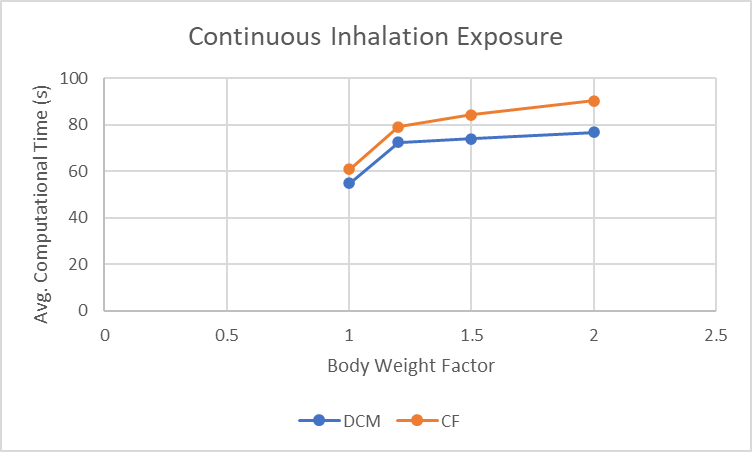

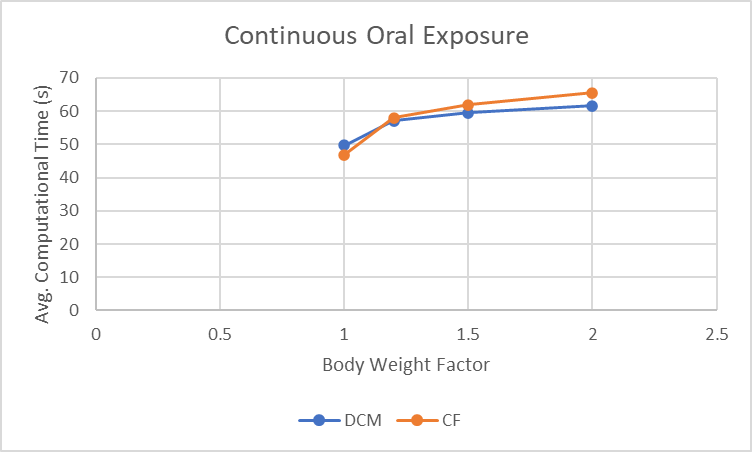

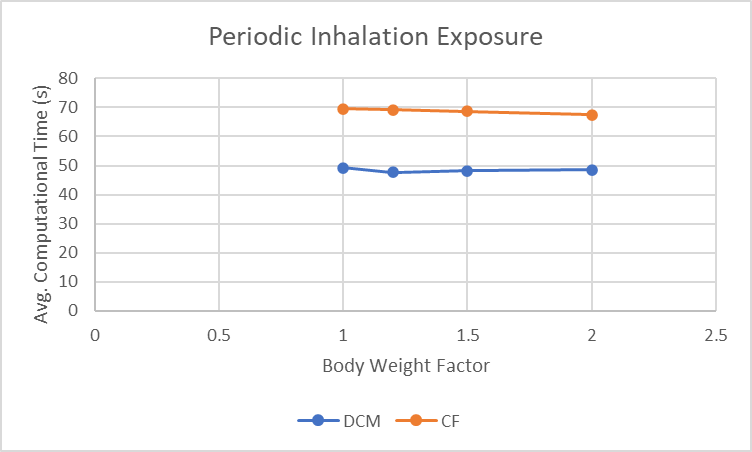

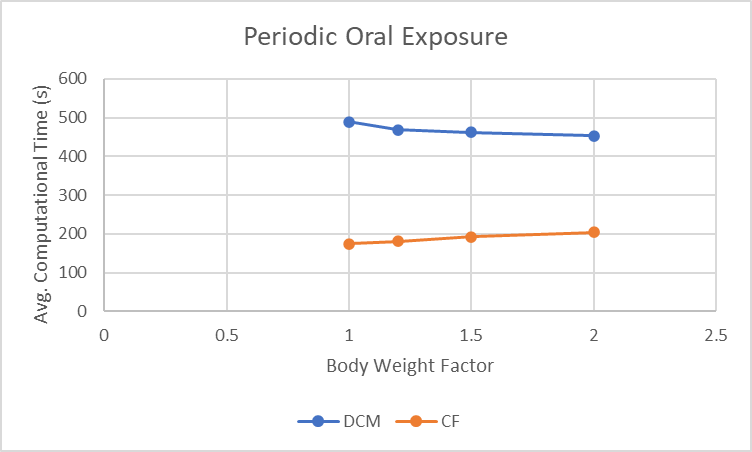


For the continuous inhalation exposures, there is an increase in computational time when using a body weight that is described by a function that changes in time, and a slight increase in time for more rapid changes in body weight. For periodic exposures, there is not a clear pattern for how a function that describes a changing body weight impacts computational time compared to a function that describes a constant body weight (at least in this limited case).
